# The Ways of the Virus: Interactions of Platelets and Red Blood Cells with SARS-CoV-2, and Their Potential Pathophysiological Significance in COVID-19

**DOI:** 10.3390/ijms242417291

**Published:** 2023-12-09

**Authors:** Mikhail A. Panteleev, Anastasia N. Sveshnikova, Soslan S. Shakhidzhanov, Alexey V. Zamaraev, Fazoil I. Ataullakhanov, Aleksandr G. Rumyantsev

**Affiliations:** 1 Department of Medical Physics, Physics Faculty, Lomonosov Moscow State University, 1 Leninskie Gory, 119991 Moscow, Russia; 2 Dmitry Rogachev National Medical Research Center of Pediatric Hematology, Oncology and Immunology, Ministry of Healthcare of Russian Federation, 1 Samory Mashela, 117198 Moscow, Russia; 3 Center for Theoretical Problems of Physicochemical Pharmacology, Russian Academy of Sciences, 30 Srednyaya Kalitnikovskaya Str., 109029 Moscow, Russia; 4 Faculty of Fundamental Physics and Chemical Engineering, Lomonosov Moscow State University, 1 Leninskie Gory, 119991 Moscow, Russia; 5 Engelhardt Institute of Molecular Biology, Russian Academy of Sciences, 32 Ulitsa Vavilova, 119991 Moscow, Russia; 6 Faculty of Medicine, Lomonosov Moscow State University, 1 Leninskie Gory, 119991 Moscow, Russia; 7 Moscow Institute of Physics and Technology, National Research University, 9 Institutskiy Per., 141701 Dolgoprudny, Russia; 8 Perelman School of Medicine, University of Pennsylvania, 3400 Civic Center Blvd., Philadelphia, PA 19104, USA

**Keywords:** SARS-CoV-2, platelet, erythrocyte, integrin, ACE-2, procoagulant platelets, neutrophil extracellular traps

## Abstract

The hematological effects of severe acute respiratory syndrome coronavirus 2 (SARS-CoV-2) are important in COVID-19 pathophysiology. However, the interactions of SARS-CoV-2 with platelets and red blood cells are still poorly understood. There are conflicting data regarding the mechanisms and significance of these interactions. The aim of this review is to put together available data and discuss hypotheses, the known and suspected effects of the virus on these blood cells, their pathophysiological and diagnostic significance, and the potential role of platelets and red blood cells in the virus’s transport, propagation, and clearance by the immune system. We pay particular attention to the mutual activation of platelets, the immune system, the endothelium, and blood coagulation and how this changes with the evolution of SARS-CoV-2. There is now convincing evidence that platelets, along with platelet and erythroid precursors (but not mature erythrocytes), are frequently infected by SARS-CoV-2 and functionally changed. The mechanisms of infection of these cells and their role are not yet entirely clear. Still, the changes in platelets and red blood cells in COVID-19 are significantly associated with disease severity and are likely to have prognostic and pathophysiological significance in the development of thrombotic and pulmonary complications.

## 1. Introduction

The first impressions of COVID-19 were associated with obvious severe pulmonary manifestations, which gave the name to the virus responsible for them. However, over a short period of several months, it became clear that the disease significantly affects not only the lungs but also many other systems in the patient’s body [1]. Among them, changes in the blood system and immune system dysregulation played a key role in the pathophysiology of disease and mortality [2]. In particular, impaired oxygen transport and the high risk of thrombotic complications attract special attention to what happens to RBCs (red blood cells) and platelets. The mechanisms of these changes are still far from being completely clear, and their understanding might be important for the development of novel diagnostic and treatment options [3].

The direct role of pathogens in hematological changes during infectious diseases is not frequently observed. Indeed, the most common acute infections prefer various mucous membranes or other tissues that are more accessible and less protected than blood, and only in extreme cases may they progress into viremia or bacteremia. When these occur, sepsis usually causes a severe and multifaceted reaction mediated by an overreaction of the immune system rather than the pathogens themselves. Though there is a category of pathogens that might even seek to get into the blood cells or deep tissues, they have special strategies to hide their presence in the blood as much as possible, and such diseases are more likely to be chronic.

It is now believed that viremia in COVID-19 is consistent with this picture; it is not obligatory in this disease, but it is not uncommon and is clearly associated with disease severity [4,5,6]. SARS-CoV-2 may use membrane-attached ACE2 (angiotensin-converting enzyme 2) to invade host cells, although a dual role of ACE2 in the disease has been proposed [7]. The most life-threatening and health-threatening features of COVID-19, such as the disseminated intravascular activation of blood coagulation, show many parallels with other types of sepsis [8]. It may not be an exaggeration to say that one of the main peculiarities of COVID-19 is that this respiratory virus is unusually active in the blood and causes viremia too often. In light of this, it is especially important to understand how SARS-CoV-2 interacts with blood and vascular cells.

Over the years, platelets and RBCs have been found to engage in numerous two-way interactions with various pathogens, including some viruses, which may have important pathophysiological consequences. Prominent examples include the binding and replication of dengue virus by platelets [9] and their progenitor cells, megakaryocytes [10], and the internalization and transport of influenza virus by platelets [11]. RBC surface proteins are capable of binding a variety of pathogens, and the role of RBCs in inhibiting bacterial phagocytosis [11] and in the transport and replication of bacteria [12] and malaria parasites is well documented. However, there is much less information about RBC interactions with viruses [13].

Pathogens can significantly impact cellular physiology, positively or negatively influencing the virus. Their direct or indirect effects play a crucial role in disease progression. Despite the fact that disturbances in the hemostasis and oxygen transport systems were early identified as striking pathophysiological features and leading causes of mortality in COVID-19 [14], the role of interactions between the virus and blood cells in the development of these disorders remains unclear [15,16].

The aim of this review is to put together the available evidence on the interactions of SARS-CoV-2 with RBCs and platelets, to highlight points of agreement and disagreement, to identify blank spots in our knowledge, and to derive a comprehensive picture of the existing hypotheses on the mechanisms of these interactions, on the hematological changes caused by them, and on their pathophysiological significance.

## 2. Two-Way Interactions of SARS-CoV-2 with RBCs and Their Precursors

There is currently little evidence that SARS-CoV-2 actively interacts with or invades mature erythrocytes, although the binding of the virus to erythrocyte proteins has been suggested [17]. One interesting possible receptor for this interaction is the Band3 anion transport protein [18], while another is the blood group system determinator basigin, also known as CD147 [19,20]. A recent study, which remains in preprint form at the moment, found an extensive association of SARS-CoV-2 with RBCs in a murine model [21]. The study also suggested an interaction with heme itself and the possible contribution of this interaction to the multi-organ spread of the virus.

On the contrary, there are quite a few indications that the virus significantly interacts with the ACE2-bearing immature erythrocyte precursors [20,22]. The COVID-19 infection of these cells dysregulates iron and hemoglobin metabolism and stimulates their reproduction [20,22]. Indeed, circulating erythroid progenitors are a characteristic feature of patients’ blood, and their concentrations are negatively correlated with hemoglobin and leukocyte levels [20,23]. This drop in hemoglobin levels, coupled with increased erythropoiesis, is considered to be one of the important mechanisms of hypoxia and respiratory problems in patients with COVID-19 [24]. Interestingly, these features are most pronounced in the original Wuhan virus and appear less in the Delta and Omicron clades [25].

**Figure 1:**
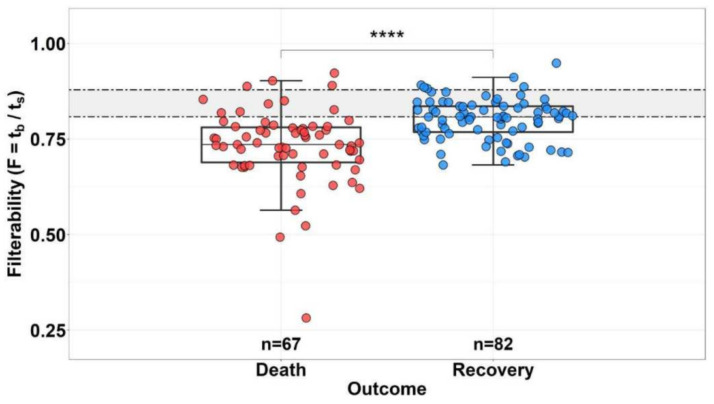
Distribution of RBC filterability in the group of deceased (*n* = 67) and survivor (*n* = 82) patients with COVID-19. Horizontal dotted lines indicate the boundaries of normal filterability values. **** The difference between the groups is significant (*p* < 0.0001). Reproduced from [27].

Patients’ RBCs are furthermore characterized by anisocytosis, a variability in sizes [26]. We have previously shown that the ability of RBCs to penetrate small capillaries (filterability) negatively correlates with the severity of the patient’s condition (Figure 1) and is a predictor of a negative prognosis [27]. It also correlates with C-reactive protein levels, suggesting an inflammatory nature of the problem. In view of the recent finding that red blood cell distribution width is associated with increased interactions of blood cells with the vascular wall [28], it is tempting to suggest linking anistocytosis with the poorly understood aspect of RBCs and their role in thrombosis and hemostasis. It is considered fairly well-established that RBCs determine blood mechanics and facilitate platelet transport, and this mechanism is vital to both hemostasis and thrombosis [29,30,31]. The altered size of RBCs in COVID-19 could therefore alter the dynamic behavior of the bulk flow and thus contribute to the platelet margination effect [32]. The ability of erythrocytes to support membrane-dependent blood coagulation reactions on their surface or to activate blood coagulation with their microvesicles has been much more controversial, but it seems noticeable, at least in pathological situations [33,34,35]. Indeed, it was found that RBCs exhibited significantly elevated apoptotic markers in the COVID-19 patients, which even correlated with D-dimer, suggesting a contribution of RBCs in the thrombotic complications of the disease [36]. Other functions of erythrocytes in thrombosis also cannot be excluded [37,38]. Although the field in general remains only marginally explored, the already available evidence and the considerations discussed make it likely that RBC changes contribute to thrombotic risks in COVID-19.

## 3. Interaction of SARS-CoV-2 with Platelets and Its Significance for the Virus

The first report of the detection of SARS-CoV-2-related RNA in platelets of patients with COVID-19 appeared in mid-2020 [39]: viral RNA was detected in platelets in approximately 20% of patients, regardless of disease severity. Around the same time, another study reported that platelets were positive for viral RNA in 6% of patients [40]. The same study reported that platelets express the major receptor for the coronavirus’s entry into different cell types, angiotensin-converting enzyme 2 (ACE2), as well as the serine protease TMPRSS2, which is important for spike protein priming. The presence of ACE2 and TMPRSS2 in platelets was confirmed by another report, which has remained a preprint [41]. Over the next three years, several additional potential receptors facilitating the binding and entry of SARS-CoV-2, apart from ACE2, were identified. These included Band3, which is expressed on both platelets and the surface of RBCs [42]. Although the presence of ACE2 in platelets and the mechanism of entry of the original coronavirus variant are not yet clear, there is already evidence that different variants of SARS-CoV-2 likely use different receptors [42], as discussed below. One interesting possibility could be CD147 [19,42].

The simpler question of the presence of SARS-CoV-2 and ACE2 in platelets has indeed been raised again and again [43]. Immunofluorescence methods are known to have low reliability and specificity when detecting low levels of antigens, and the majority of proteomic and transcriptomic studies in 2020–2021 could not identify ACE2 in platelets [44]. For example, an in-depth study of platelet gene expression and function in COVID-19 failed to detect ACE2 in platelets, either RNA or protein [45]; viral RNA was detected only in two out of 25 patients. However, a comparison of methodologies showed that the result was highly dependent on the method: while PCR detected viral RNA in a minority or none of the patients, RNA-sequencing showed the presence of fragments of the viral genome in all patients [46]. Finally, transmission electron microscopy does detect viral particles in the patient’s platelets [47].

Given these data, it can be tentatively concluded that platelets from patients with COVID-19 are, after all, likely to contain SARS-CoV-2, although the mechanism of entry is not certain. What could be the consequences of the virus infection? The possibilities discussed in the context of a pathogen in blood cells are defense against the immune system, exposure or presentation to the immune system attack, transport and transmission to other tissues, and replication (which is difficult for a virus in platelets and RBCs). The in vitro incubation of platelets and MEG01 megakaryocyte-like cells with SARS-CoV-2 confirmed the ability of both cell types to slowly engulf the virus without replicating it [48]. This study did not detect ACE2 or TMPRSS2 in platelets. Increased autophagy markers in platelets from COVID-19 patients and their co-localization with coronavirus proteins suggest that platelets digest the virus through the xenophagy process [47]. On the other hand, changes in the differentiation and expression of antiviral proteins in circulating megakaryocytes from patients with COVID-19 and evidence that megakaryocytes can become infected with the virus through infected platelets [49] suggest that platelets may contribute to the spread of the virus and the development of negative changes in systemic circulation. Considering that the lungs are among the potential sites of thrombocytopoiesis, the presence of infected megakaryocytes in this area could potentially lead to direct respiratory invasion. 

## 4. Platelets and Endothelium: Ways of Interaction

It is now believed that platelets play not one but two vital roles in maintaining the integrity of the vascular wall. First, they are involved in repairing damage caused by traumatic injuries. This process occurs under conditions of high flow rates and requires the formation of a hemostatic plug [50]. Second, they support the homeostasis of the endothelium and the tightness of intercellular contacts, which is achieved by single platelets without the formation of a hemostatic plug [51]. This function may be especially relevant during coronavirus infection.

Although the traditional view focuses on platelet adhesion and activation related to the proteins of the intercellular matrix, primarily collagens, it has long been known that platelets are capable of direct adhesion to endothelial cells [52]. In healthy vessels, platelet–endothelial interaction is prevented both by the presence of endogenous platelet inhibitors (NO and prostacyclin) and by the absence of platelet adhesion sites on the endothelial surface. This picture changes radically with micro-damage, inflammation, or the presence of other cells on the vessel wall, e.g., immune or cancer cells. In these cases, the main adhesion bridges are pairs P-selectin (active platelets)-PSGL-1 (inactive/active endothelium), GP1b (platelets)—vVW (active endothelium), integrins (active platelets)—fibrinogen—integrins (active endothelium), and CLEC-2 (platelets)—podoplanin (active endothelium) [53]. A separate case is the interaction of platelets with fibrin when the activated endothelium exposes tissue factor [52]. As seen from the list above, platelet adhesion requires either their activation or the activation of the endothelium. In the case of micro-damage, platelet activation can occur due to a certain amount of ADP from damaged cells. In the case of inflammation, it could be caused by interactions between glycoprotein Ib and von Willebrand factor or cytokines secreted by immune cells [54].

It should be further noted that, with the exception of platelet adhesion to vVW, all other cases of the adhesion and activation of platelets at the site of adhesion are likely to result in the secretion of the contents of their granules. It is unknown what role this process plays in endothelial physiology; however, platelet-derived growth factors are well known to promote the development of vascular tumors, vascular growth, and vascular cell proliferation [55]. There is evidence that platelets normally reduce the permeability of the endothelium to albumin [56], while, during inflammation, activated platelets increase the permeability of the vascular endothelium [57]. Thanks to this, the virus from the blood can enter the endothelium and sub-endothelial layer.

Although platelets have long been considered important for maintaining the health of the vascular wall, the first experimental and molecular evidence of the protective role of platelets appeared in 2008, when it was shown how inflammation causes bleeding in thrombocytopenia [58]. It is now quite reliably established that this function of platelets does not require the formation of an aggregate. How exactly bleeding (which platelets protect against) occurs depends on the specific location and associated factors. For example, in most cases, hemorrhage is associated with the transmigration of leukocytes. This further complicates an understanding of the role of platelets, since leukocyte recruitment also requires platelets. 

Unlike hemostasis and thrombosis, which require approximately the same set of platelet functions, preventing endothelial damage requires different sets of functions in different organs [51]. For example, hemostasis and thrombosis always fundamentally require GPIb (for primary platelet attachment) and GPIIb-IIIa (for the stabilization of aggregates). This is not the case for vascular wall integrity: GPIb is important for the maintenance of pulmonary and cerebral vascular endothelium in ischemic stroke (but not in the ischemia-reperfusion model). GPIIb-IIIa is important for brain and lung endothelium in most models but not cutaneous hemorrhage. In contrast, GPVI and CLEC-2 appear to be less important in hemostasis but consistently appear to be involved in both endothelial protection and inflammatory bleeding. We have a fairly poor understanding of how platelets in general carry out their defense, but recent work on autonomous platelet migration may provide a clue [59].

Moreover, three years ago, it was shown that platelets can be targeted at endothelial junctions due to the fibrin gradient [60]. Platelets can then release numerous molecules that drive the local endothelial reprogramming and healing of micro-damage. This pathway is crucial for maintaining vascular homeostasis. It may be involved in both the protective functions of platelets during COVID-19 and potentially aiding virus spread. However, it is unlikely to easily lead to the entry of the virus from the cytoplasm, especially if the hypothesis of its association with mitochondria, as described below, is correct.

To what extent can viruses enter the endothelium through the direct uptake of material from platelets? The topic of platelet absorption by endothelial cells is quite controversial, although there is evidence in favor of some mechanisms of this kind [61]. The endocytosis of platelet microvesicles by endotheliocytes is much better documented [62]. This leads to the entry of platelet contents into endothelial cells, including both endothelium-reprogramming miRNA [63] and entire mitochondria [64]. In this regard, the possibility of the endocytosis of platelet microvesicles with SARS-CoV-2 by endothelial cells seems promising because this is a direct route of infection of the endothelium without the participation of receptors discussed when considering the problems of hemostasis. Given the recently demonstrated ability of platelet microvesicles to penetrate bone marrow and influence megakaryocytes [65], which is definitely an attractive opportunity for a virus, this mechanism cannot be ruled out.

## 5. How SARS-CoV-2 Affects Platelets

**Figure 2:**
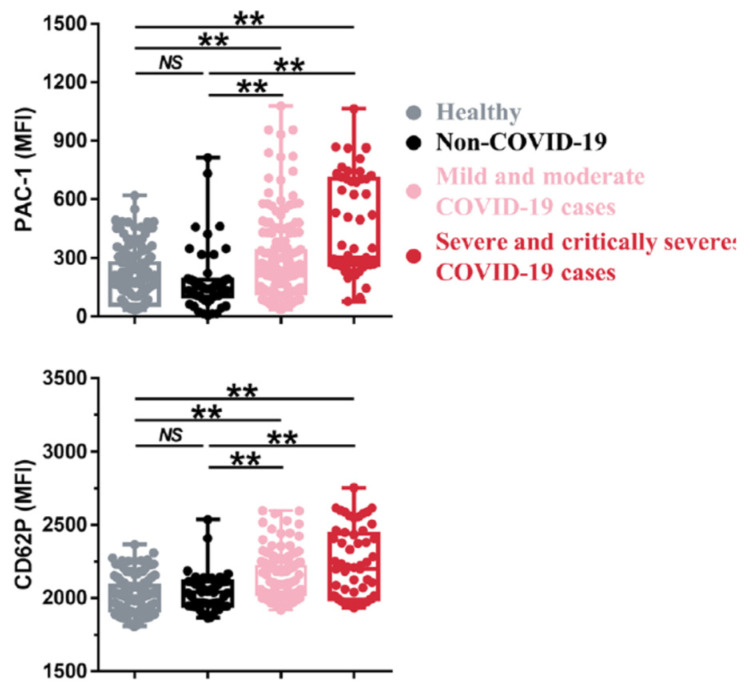
Platelet functional parameters in COVID-19 patients. The panels show mean fluorescence intensity for antibodies against activated integrin αIIbβ3 (**top**) and P-selectin (**bottom**) in a cohort of COVID-19 patients (*n* = 241). NS, not significant; **, *p* < 0.05. Reproduced from [40], published by a BMC Journal of Hematology & Oncology.

The very early studies of platelets in COVID-19 revealed the presence of their hyperactivation in patients [39]. Over the next two years, this picture was confirmed and refined. Patients’ platelets circulate in a partially activated state (Figure 2), exhibiting increased size, elevated levels of alpha granule markers, and the activation of integrins and phosphatidylserine in the outer layer of the membrane [39,40,45,66,67,68,69,70,71]. Some of these activation markers overlap with the markers of apoptosis, which is not always easy to differentiate [46]. Numerous platelet aggregates with neutrophils, monocytes, and T cells are regularly detected in the blood of patients [45,67,70,72,73,74]. Thrombocytopenia with COVID-19 is not necessary but is quite common [75].

The picture of platelet response to stimulation is more complex and contradictory. While some studies suggest that patients’ platelets display increased aggregation, adhesion, secretion, and clot formation [39,45,68], others show refractoriness and weakened responses [66,67,69]. This problem may be related both to the selection of patient groups and to the methodological difficulties of studying platelet responses. Even in the case of the more reproducible and stable condition of immune thrombocytopenia, a comparable level of uncertainty has persisted over an extended period. While most studies concur that platelets in immune thrombocytopenia circulate in a partially activated state [76,77,78,79], certain methodologies report a weakened response to stimulation [77,78], while others denote it as normal or heightened [76,79,80], and still others note a heterogeneity of responses among different patient groups [81].

Some studies indicate a connection between certain markers of platelet hyperactivation and the severity of the disease. Interestingly, in one of the first studies, an increase in phosphatidylserine-positive platelet vesicles was observed in patients with moderate form [39]. Other work indicates that an increased volume and level of activation of platelet integrins are positively correlated with disease severity [40,68,71]; an association has also been reported for levels of circulating aggregates with complications or disease severity [70,72]. Interestingly, the previously cited work [49] found the virus in the platelets of almost all non-survivors but not the survivors. Other studies have not observed a strong correlation between platelet quality and disease severity or outcome, except in a group of patients on extracorporeal membrane oxygenation (ECMO) [66].

ECMO is an important independent factor for platelet dysfunction in COVID-19. Platelet activation and subsequent refractoriness in a wide variety of patients on ECMO were observed long before the pandemic [82,83,84]. However, COVID-19 itself alters platelet function, and ECMO in its severe cases is very common. The observation of severe thrombocytopenia and platelet dysfunction identified on ECMO in COVID-19 indicates that it might play a role in the negative outcome and may be a target for therapy [66].

**Figure 3:**
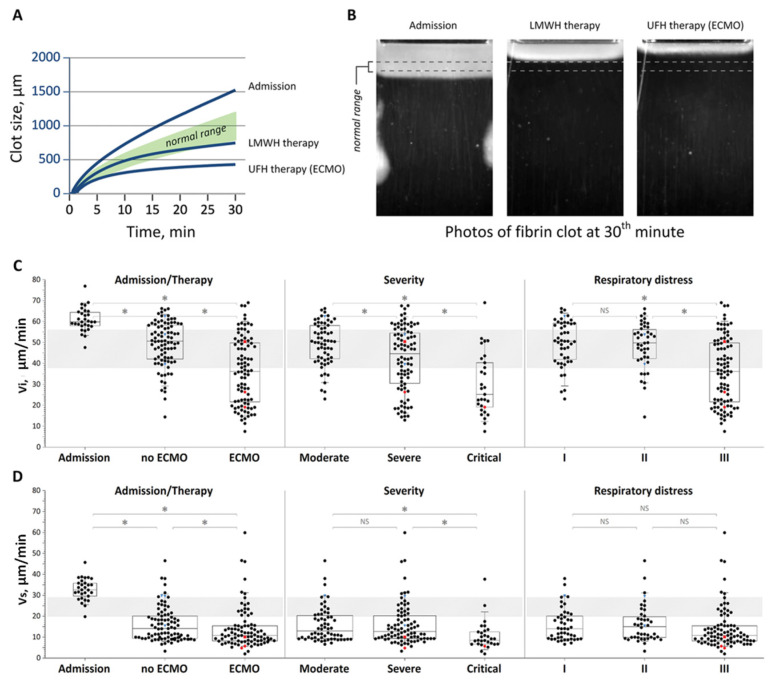
Thrombodynamics assay parameters in the COVID-19 patients grouped based on the severity and treatment stage. (**A**) Typical curves of clot size as a function of time, and (**B**) typical clot images for the patient groups. Panels (**C**) and (**D**) show initial and stationary clot growth rates Vi and Vs, respectively. NS, not significant; *, *p* < 0.01. Red symbols indicate bleeding epizodes, blue symbols indicate thrombotic episodes. Reproduced from [8].

How does the coronavirus itself manage to affect platelets? Some studies suggest that platelets can be activated by viruses directly [40,46], including the MAP kinase pathway of intracellular signaling associated with ACE2 [27,45]. Another hypothesis is that platelet activation is secondary to systemic intravascular coagulation [66], which is well documented in coronavirus disease (Figure 3). In support of this idea, treatment with increased cumulative doses of heparin was significantly associated with improvements in platelet parameters [66]. This is also indicated by data from longitudinal studies, which show a lack of correlation of platelet status with disease dynamics and viremia and a very slow normalization of their status in recovering patients compared to parameters of inflammation and viremia [66]. There is little data on the effect of antiviral treatment on platelet status [85,86]. 

It can be concluded that platelet preactivation in circulation and the formation of circulating platelet aggregates with other cell types appear to be reliably established in COVID-19. The pathophysiological significance of these changes has not been well established yet. The most recent meta-analyses of multiple clinical trials indicate that antiplatelet drugs provide little if any benefit in COVID-19 [87], although this may be because existing drugs focus on the wrong platelet functions. The mechanisms of platelet activation in the circulation, the functions of preactivated platelets, the association of altered platelet parameters with the severity of the disease, and their applicability for predicting outcomes and adjusting therapy require further investigation.

## 6. Platelet Subpopulations and Mitochondria in COVID-19

The appearance of phosphatidylserine on the outer leaflet of the platelet plasma membrane differs from “normal” activation and requires a separate discussion since it is associated with the transition of platelets to a special procoagulant state. Procoagulant platelets, a hyperactivated subpopulation with high procoagulant activity typically detected when strongly activated by thrombin, collagen, or a combination of both, are an intriguing mystery in the physiology and pathology of the hemostatic system. It is believed that their formation occurs through the mechanism of mitochondrial necrosis [88], as a consequence of overloading some mitochondria with calcium [89]. Although they are not pre-existing and are formed only during activation, factors such as baseline cytosolic calcium [90] or mitochondrial number [91] may predispose a platelet to become procoagulant. Procoagulant platelets do not have active integrins but carry a “coat” or “cap” of alpha granule proteins entangled and cross-linked by transglutaminases in a mesh of fibrin [92], which allows them to incorporate into thrombi [93]; they are also capable of interacting with cells of the immune system [94]. Procoagulant platelets are normally rapidly cleared from the circulation but may be elevated in patients with a systemic activation of hemostasis: typical examples include immune thrombocytopenia [76] and pre-term infants [95].

Increased levels of procoagulant platelets in COVID-19 [66,96] may play an important role in the thrombotic complications associated with this disease. In addition, due to their peculiarities in adhesion surface proteins and cell–cell interactions [94], procoagulant platelets may play additional roles in viral transport or, conversely, promote virus uptake by immune cells. Interestingly, the level of activation of platelet caspase-1 and Bruton’s tyrosine kinase, which are involved in some of the possible pathways for the formation of procoagulant platelets, correlates with the severity of the disease [97]. Conversely, those who recovered from mild COVID-19 do not have a procoagulant platelet phenotype, and their plasma does not activate platelets from healthy donors [98].

What exactly causes procoagulant platelets to appear? The simplest hypothesis is that it is, in parallel with “normal” activation, induced by thrombin as a consequence of systemic intravascular coagulation [66]. On the other hand, there is evidence that a fraction of immunoglobulins in patient plasma is capable of inducing the formation of procoagulant platelets [96]. This raises interesting associations with the platelet phenotype in ITP (immune thrombocytopenia) [76,99], where preactivation is associated with autoantibodies. Another proposed mechanism for promoting the formation of procoagulant platelets by the patient’s immune system is the ability of calprotectin S100A8/A9 (a myeloid cell-secreted antimicrobial and proinflammatory alarmin that is elevated in COVID-19) to induce the release of phosphatidylserine in platelets [100]. Finally, the fourth mechanism is the ability of the SARS-CoV-2 virus Spike protein to directly activate the key chloride channel and platelet phospholipid scramblase TMEM-16F and thereby induce the formation of procoagulant platelets [101]. Identifying which of these mechanisms of procoagulant platelet induction actually play the leading role may be critical for selecting therapy for the disease. For example, the first mechanism could be easily controlled by anticoagulant therapy, but it should not directly affect any of the other three.

In any case, the formation of procoagulant platelets occurs through the mechanism of cell death and is mediated by mitochondria [89,90,91]. Their role in the platelet is poorly understood since all estimates indicate that the glycolytic pathway is sufficient to meet all platelet energy needs. We know that coronavirus RNA appears to colocalize with mitochondria [102,103]. There is much speculation about how exactly mitochondria help the coronavirus replicate and evade the immune response, but there is little concrete data [104,105,106]. On the other hand, there are quite a lot of studies showing that mitochondria are disrupted during COVID-19 in platelets [107], leukocytes [108], microglia [109], and cardiomyocytes [110]. In most cases, this is discussed in the context of the long-term consequences of the disease. However, given the role of platelet mitochondria in the emergence of procoagulant platelets [89,90,91], along with their subsequent vesiculation and the potential importance of their transfer to endothelial cells [64], the involvement of this phenomenon in the acute phase processes of the disease cannot be excluded.

## 7. Evolution of Coronavirus and Its Hematological Interactions

As noted above, different variants of the SARS-CoV-2 coronavirus may differ significantly in their characteristics of certain interactions. Below we shall briefly formulate the known data on the evolution of the virus in order to attempt puzzling together the data on the differences in its hematological manifestations and interactions.

The original Wuhan variant of SARS-CoV-2 changed little during the first half of 2020, but then new variants began to emerge rapidly [111]. It is now believed that the main driving forces behind this accelerated evolution were the development of neutralizing antibodies in the population after the first wave, followed by large-scale immunization. However, selection based on proliferation rate and ability to penetrate target cells may also have been important. The vast majority of mutations beneficial to the virus were associated with its main Spike protein, which is responsible for ACE2 binding [112].

The first milestone in the evolution of the coronavirus was the D614G mutation in the Spike protein, which contributed to increased cell penetration and infectivity. Interestingly, this mutation was stepped down rather than up from the point of view of avoiding immunity [111]. This genetic branch (clade) of the virus was not designated as a VOC (variant of concern) in the World Health Organization classification at the time. However, it is presently considered fundamental [113].

The first official VOC was Alpha, which emerged in the second half of 2020 in England [114] and acquired the E484K mutation in the same Spike protein [115]. Its ability to transmit between people was 50% higher than that of the wild type virus [116]. The Beta and Gamma variants emerged later in 2020 in South Africa and Brazil, respectively, and differed by mutations at positions E484 and K417. As a result, all variants of SARS-CoV-2 with combinations of mutations N501Y, E484K, K417N/T had impressively reduced sensitivity to neutralizing antibodies [115].

These regionally successful variants were followed in December 2020 by the Delta clade, which emerged in India and became dominant globally by mid-2021. The radical step (which appears to have determined its success) was the P681R mutation in the same protein at the serine proteinase cleavage point. It unexpectedly turned out to be useful for fusogenicity: the ability of the virus to fuse infected cells together with neighbors, forming a giant cell, a syncytium. In general, the fusogenicity of the strains steadily increased with evolution and reached a maximum in the Delta strain. Quantitative estimates show that its ability to transfer between people was also almost twice that of Alpha [116]. Delta did not have any advantages in its ability to evade neutralizing antibodies. This has changed in the Omicron clade, which completely overhauled the Spike protein [117].

It appears to have emerged in November 2021 in Botswana and South Africa, and its variants have since become dominant worldwide [115]. They carry dozens of different mutations in the Spike protein [111], much more than any previous VOC. Omicron’s ability to evade antibodies against previous clades has improved dramatically. The downside of the multiple mutations is a marked reduction in infectivity and fusogenicity. The ongoing evolution of Omicron over the past couple of years has spawned a large number of sub-variants. Some of them had improved abilities to infect cells, fusogenicity, and suppress immunity, but so far, any sub-variants of Omicron have been inferior in fusogenicity to any of the pre-Omicron strains.

Clinical data on the hematological manifestations of the different clades are not complete. As noted above, the Wuhan variant resulted in a greater number of circulating CD71+ erythroid progenitors with immunosuppressive properties compared with Delta and Omicron [25]. Epidemiologically, Omicron is considered several times less lethal and has weaker manifestations of acute and post-Covid problems than Delta, but is highly contagious and has a high chance of re-infection [115]. In general, Omicron moves from the lungs to the bronchi [118]. The frequency of thrombotic events in the Delta strain was 35% versus 25% in previous strains [119]. The same was shown for the lesser-known Gamma variant [120]. The incidence of acute limb ischemia doubled with the onset of Delta [121]. An analysis of post-Covid arterial thrombosis in Indian patients revealed an incidence of 4% in Alpha, 15% in Delta, and less than 2% in Omicron [122].

Over the last several months, a wave of studies has focused on the experimental modeling of platelet activation by variants of the Spike protein. The first study showed that the spike protein can bind integrins αIIbβ3 (glycoprotein IIb-IIIa) and activate platelets, with Kappa and Delta having stronger effects relative to the wild type [123]. Protein–protein interaction studies revealed that platelet integrins bind to the RGD sequences in the Spike protein of Alpha but not wild type or Omicron [124]. Another study of platelet interactions with SARS-CoV-2 spike protein variants found no effects [125]. Yet another found significant effects on platelet activation, with the greatest effect in Delta, slightly weaker in Alpha, with wild type and Omicron tied for last place [126]. The next work [127] showed that the wild type caused a threefold increase in integrin activation and P-selectin expression compared to the resting state, Delta activated them five–sixfold, and Alpha and one of the Omicron subtypes less than twofold (but another Omicron subtype was close to the wild type). As discussed above, Delta was also superior to the Wuhan variant in activating neutrophils.

It seems that three main stages of hematological manifestations can be distinguished: the Wuhan strain and its pre-Delta versions, the increasingly thrombogenic Delta, followed by the milder Omicron. Thrombogenic ability appears to correlate with the ability of the virus to be fusogenic and aerobic, with the ability of its spike protein to bind the ACE2 receptor, activate neutrophils, bind platelet integrins, and activate platelets. Given the propensity of platelets described above to settle at endothelial cell junctions, one can also cautiously speculate that this may also contribute to fusogenicity. As another tendency, the ability to damage RBCs and suppress immunity through erythroid precursors has been steadily dropping at the Delta level. Unfortunately, we do not have data to relate these observations with the problems discussed above, such as the ability of the virus to be carried by platelets or penetrate the endothelium.

## 8. Endothelium, Blood Coagulation, Neutrophils and SARS-CoV-2

In connection with the above considerations, it seems important to return to the topic of the endothelium. This time, we should consider it from the opposite point of view: how it directly and indirectly (through blood coagulation) affects the pathophysiology of COVID-19. It is important to emphasize that a striking feature of the pathogenesis of COVID-19 is damage to the blood vessels of the organs that have a special microvascular network: lungs, brain, kidneys, and bone marrow. This is inextricably linked with thrombohemorrhagic syndrome, which is extremely difficult to correct and is one of the main causes of death in severe patients. In fact, the question of the nature of thrombohemorrhagic syndrome (and disseminated intravascular coagulation) in sepsis of any nature is very complex, but modern opinion is inclined to believe that the direct initiator of coagulation is the tissue factor on the inflamed endothelium [128], although some bacteria are capable of activating blood clotting and platelets by themselves.

Damage to the endothelium by the virus through ACE2 in patients with COVID-19 became obvious in 2020 [129]. Interestingly, experiments in genetically modified mice show that systemic SARS-CoV-2 infection is most pronounced locally, with platelet and neutrophil aggregates and endothelial barrier disruption observed in the brain and lungs [130]. It can be assumed that interactions of the virus with platelets and their precursors may play a role in infection and then damage to the endothelium in these places. This infection, in turn, leads to endothelial activation and disseminated blood coagulation, which then further activates platelets. At the same time, the interaction of the virus with erythrocyte precursors leads to defects in the functioning of erythrocytes, manifested in the development of systemic hypoxia and potentially aiding coagulation.

Another option for activating coagulation is through extracellular traps of neutrophils. The increased formation of neutrophil extracellular traps has been reported in the blood of patients [66]. Platelets are able to stimulate neutrophil activation and NET (neutrophil extracellular trap) formation, and vice versa [131]. In addition to activating coagulation, the NETs lead to the development of inflammation, epithelial–mesenchymal transition, and fibrosis in the lungs of patients [132]. The level of NETosis correlates with the severity of the disease [133]. There is evidence of the direct activation of neutrophils by the Spike protein, especially the Delta variant [134]. It has also been shown that macrophages can bind the Spike protein, leading to neutrophil recruitment and damage [135]. This variant was further enhanced by the direct stimulation of neutrophils with the Spike protein.

**Figure 4:**
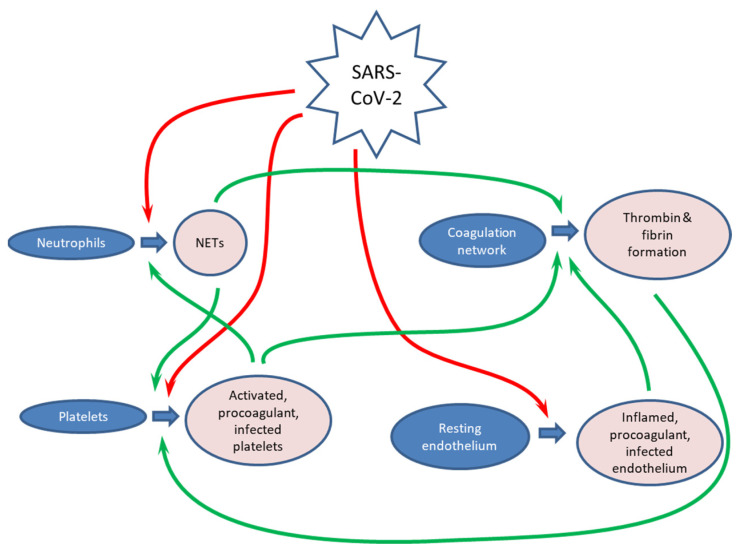
The network of SARS-CoV-2 interactions with blood cells. Red arrows show first-level effects of the virus on the target cells and systems, while green arrows indicate second-level and feedback activation, when these affected cells and systems further stimulate each other. The figure was created using Microsoft PowerPoint.

Thus, the activation of coagulation, platelets and neutrophils seem to continuously reinforce each other in COVID-19 according to the following pattern (Figure 4):

(1) The Spike protein activates platelets and neutrophils directly, and the virus also damages the endothelium leading to blood clotting.
(2) Neutrophil extracellular traps activate platelets and blood clotting.
(3) Platelets activate neutrophils in positive feedback and accelerate or even activate [136] blood clotting through their procoagulant activity.
(4) Blood clotting acts as a nexus in the network, as it is activated or accelerated by all three cell types. It further activates platelets in positive feedback. It also acts as the final executor in the system, as this activation of the coagulation cascade ultimately appears to be the immediate cause of increased thrombotic risks and organ failure in COVID-19.

## 9. Problems and Their Status

To better illustrate the current state of the art, we summarized it in a systematic table (Table 1), which includes all alternative hypotheses for each issue and highlights their status at present.

The presence of SARS-CoV-2 has been remaining a point of controversy for quite some time, but the recent evidence with highly sensitive RNA assays and electron microscopy [46,49,137] strongly tilts the balance towards the view that SARS-CoV-2 is present in the platelets of a significant fraction of patients or even the majority, especially with severe forms of COVID-19. Importantly, in addition to platelets themselves, their precursors may be an important target of the virus.

On the contrary, contradictions remain on the subject of how exactly it goes there. The canonic ACE2 entry has not yet been either proven or ruled out, and most likely there are alternative entry routes in any case [19,25,40,41,44,48,124]. It is interesting to note that the early cell-based studies on SARS-CoV-2 entry mechanisms [40,41,42,45,48] and recent studies on platelet interactions with recombinant Spike proteins focusing on platelet activation [123,124,125,126,127] appear to represent two different strategic approaches with different results. Their combination might shed light on both issues of entry and platelet activation by the virus.

While the virus cannot replicate in platelets, we cannot exclude the possibility of its exit from platelets, either directly or through the absorption of platelets and their microparticles by other cells [49]. The alternative possibility that the interaction with platelets has the opposite significance and actually contributes to the neutralization of the virus also has solid evidence behind it [47], and this requires further research.

Going back to the more reliable aspects, systematic changes in platelet function in COVID-19 can be considered well established. The researchers generally agree that thrombocytopenia is common but not severe (unless ECMO is used) and that platelets circulate in a pre-activated state, with at least some of them in an “over-activated” procoagulant (necrotic) state [39,40,45,66,67,68,69,70,71], and some in aggregates with other cells [45,67,70,72,73,74].

Nonetheless, the mechanisms of this activation are still being determined and may include direct interactions with the virus (most likely via integrins), secondary activation by blood clotting, or secondary activation by the immune system. This subject is extremely unclear and strongly clade-dependent. We might carefully speculate that all these pathways are real and possible, but identifying the significance of each requires an extremely comprehensive study, complicated all the way by ongoing viral evolution. Still, such identification may turn out to be a clue for innovative treatment approaches whose significance goes beyond COVID-19. 

There are reasons to believe that these platelet changes play a pathophysiological role, at least in the increased risk of thrombus formation in COVID-19. Given the recent breakthrough in the role of platelets in the regulation of vascular integrity, immunity, and cell proliferation, the role of platelet–virus interactions in other aspects of pathophysiology, including inflammatory positive feedback or syncytium formation, cannot be excluded. However, extensive clinical studies are required to reliably use this for diagnostic or therapeutic purposes.

Unlike platelets, mature RBCs are not likely to be an attractive target for SARS-CoV-2, but erythroid precursors are. As a result of this (and also, most likely, of the general state of the disseminated activation of hemostasis and cytokine storm), the erythrocytes of patients are significantly changed. They circulate in an immature form, carry little hemoglobin, are heterogeneous, and have filterability defects. All of these are likely to play an important role in the pulmonary problems of COVID-19 and may also contribute to other pathophysiological aspects, first of all thrombosis. The diagnostic significance of changes in patients’ erythrocytes already seems to be reliably established, while the possibility of specific therapy correction deserves the most serious discussion. 

A critical aspect of these issues that is now coming to the fore as the virus evolves is the influence of the clade. It is the most poorly studied, but there is a feeling that the pathophysiologically critical interactions of SARS-CoV-2 with blood cells have changed very much in the newer clades compared to the classic Wuhan virus. We see that some of the pathological aspects of the disease disappear as mutations occur. On the one hand, this improves the prognosis; on the other hand, some of the drugs that were important in the early stages of the pandemic may become useless or even harmful.

## 10. Conclusions

RBC progenitors, but not RBCs, are infected by SARS-CoV-2, which affects RBC quality with important consequences for oxygen transport and, probably, thrombosis. Platelets and their precursors are both infected by SARS-CoV-2 and are activated by it directly or indirectly. This is likely to contribute to thrombosis and inflammation and may also affect virus transport and recognition by the immune system.

## Figures and Tables

**Table 1:** Problems and their status.

Problem	Possibilities	Current Status
Presence of SARS-CoV-2 in platelets	1. Usually present 2. Present in a significant proportion of patients 3. Present in a subgroup of patients 4. Does not enter platelets	Likely present in a significant proportion of patients
Putative receptors and other molecules involved in platelet binding, entry, and possible transmission of virus by platelets.	ACE2, TMPRSS2, CD147, NRP-1, CD26, AGTR2, Band3, KREMEN1, ASGR1, ANP, TMEM30A, CLEC4G, LDLRAD3, GPIIb-IIIa	Not clear
Ability to replicate in platelets	1. Can 2. Cannot	Not likely
The ability to escape from platelets, or to infect through the absorption of platelets by other cells along with the virus	1. Can 2. Cannot	Not clear
The significance of the presence of virus in platelets for the virus	1. The platelet protects the virus from the immune system 2. The platelet facilitates the transport of the virus. 3. The platelet destroys viruses or contributes to its damage by the immune system.	Not clear
Pathophysiological significance of the presence of the virus in platelets for hemostasis and the body as a whole	1. Affects important functional changes in platelets 2. Does not affect, or the effect is not clinically significant	Not clear
Diagnostic significance of the presence of the virus in platelets	1. It allows for a prediction of the course of the disease and an adjustment in therapy. 2. It has no prognostic role.	Most studies point to a link between the presence of the virus in platelets and more severe forms of the disease. There is no evidence of successful therapy correction.
Platelet functional status in COVID-19	1. Preactivated/at rest 2. Present/absent procoagulant subpopulation 3. Functional responses improved/deteriorated	1. Pre-activated 2. The procoagulant subpopulation is present 3. The functional status of platelets is not clear
Mechanism of platelet activation in COVID-19, mechanism of procoagulant platelet formation	1. Directly by virus (ACE2 candidates, TMEM-16F) 2. Secondarily, through blood coagulation 3. Secondarily, through the immune system (immunomodulins, alarmins)	Not clear
Pathophysiological significance of platelet functional changes	1. Contributes to thrombosis in COVID-19. 2. Contributes to the spread of the virus or other aspects of the disease. 3. There is no pathophysiological significance.	Pathophysiological significance is more likely to exist, but the mechanisms need to be clarified.
Diagnostic significance of platelet functional changes	1. Platelet function can be a prognostic biomarker. 2. There is no direct connection.	There are contradictions. ECMO appears to be an important independent factor in the alteration of function.
Interaction of the virus with RBCs	1. Interacts with mature RBCs 2. Does not interact	There is no data on such interactions
Interaction of the virus with erythroid precursors	1. Interacts 2. Interacts and influences 3. Does not interact	Rather, it interacts, presumably through ACE2. Stimulates reproduction.
Pathophysiological and diagnostic significance of the effect on erythrocytes	1. Appearance of immature forms of erythrocytes. 2. Anisocytosis 3. Filtration defects	Pathophysiologically significant, there are associations of erythrocyte status with disease severity and prognosis.

## Data Availability

Not applicable.

## References

[1] Giovanetti M., Branda F., Cella E., Scarpa F., Bazzani L., Ciccozzi A., Slavov S.N., Benvenuto D., Sanna D., Casu M. (2023). Epidemic history and evolution of an emerging threat of international concern, the severe acute respiratory syndrome coronavirus 2. J. Med. Virol..

[2] Al-Samkari H., Karp Leaf R.S., Dzik W.H., Carlson J.C., Fogerty A.E., Waheed A., Goodarzi K., Bendapudi P., Bornikova L., Gupta S. (2020). COVID and Coagulation: Bleeding and Thrombotic Manifestations of SARS-CoV-2 Infection. Blood.

[3] Kabir M.T., Uddin M.S., Hossain M.F., Abdulhakim J.A., Alam M.A., Ashraf G.M., Bungau S.G., Bin-Jumah M.N., Abdel-Daim M.M., Aleya L. (2020). nCOVID-19 Pandemic: From Molecular Pathogenesis to Potential Investigational Therapeutics. Front. Cell Dev. Biol..

[4] Jacobs J.L., Bain W., Naqvi A., Staines B., Castanha P.M.S., Yang H., Boltz V.F., Barratt-Boyes S., Marques E.T.A., Mitchell S.L. (2022). Severe Acute Respiratory Syndrome Coronavirus 2 Viremia Is Associated With Coronavirus Disease 2019 Severity and Predicts Clinical Outcomes. Clin. Infect. Dis..

[5] Giacomelli A., Righini E., Micheli V., Pinoli P., Bernasconi A., Rizzo A., Oreni L., Ridolfo A.L., Antinori S., Ceri S. (2023). SARS-CoV-2 viremia and COVID-19 mortality: A prospective observational study. PLoS ONE.

[6] Lawrence Panchali M.J., Kim C.M., Seo J.W., Kim D.Y., Yun N.R., Kim D.M. (2023). SARS-CoV-2 RNAemia and Disease Severity in COVID-19 Patients. Viruses.

[7] Behl T., Kaur I., Bungau S., Kumar A., Uddin M.S., Kumar C., Pal G., Sahil, Shrivastava K., Zengin G. (2020). The dual impact of ACE2 in COVID-19 and ironical actions in geriatrics and pediatrics with possible therapeutic solutions. Life Sci..

[8] Bulanov A.Y., Bulanova E.L., Simarova I.B., Bovt E.A., Eliseeva O.O., Shakhidzhanov S.S., Panteleev M.A., Roumiantsev A.G., Ataullakhanov F.I., Karamzin S.S. (2023). Integral assays of hemostasis in hospitalized patients with COVID-19 on admission and during heparin thromboprophylaxis. PLoS ONE.

[9] Simon A.Y., Sutherland M.R., Pryzdial E.L. (2015). Dengue virus binding and replication by platelets. Blood.

[10] Vogt M.B., Lahon A., Arya R.P., Spencer Clinton J.L., Rico-Hesse R. (2019). Dengue viruses infect human megakaryocytes, with probable clinical consequences. PLoS Negl. Trop. Dis..

[11] Koupenova M., Corkrey H.A., Vitseva O., Manni G., Pang C.J., Clancy L., Yao C., Rade J., Levy D., Wang J.P. (2019). The role of platelets in mediating a response to human influenza infection. Nat. Commun..

[12] Deng H., Pang Q., Zhao B., Vayssier-Taussat M. (2018). Molecular Mechanisms of Bartonella and Mammalian Erythrocyte Interactions: A Review. Front. Cell Infect. Microbiol..

[13] Pretini V., Koenen M.H., Kaestner L., Fens M., Schiffelers R.M., Bartels M., Van Wijk R. (2019). Red Blood Cells: Chasing Interactions. Front. Physiol..

[14] Kipshidze N., Dangas G., White C.J., Kipshidze N., Siddiqui F., Lattimer C.R., Carter C.A., Fareed J. (2020). Viral Coagulopathy in Patients With COVID-19: Treatment and Care. Clin. Appl. Thromb. Hemost..

[15] Kosenko E., Tikhonova L., Alilova G., Montoliu C. (2023). Erythrocytes Functionality in SARS-CoV-2 Infection: Potential Link with Alzheimer’s Disease. Int. J. Mol. Sci..

[16] Zhao J., Xu X., Gao Y., Yu Y., Li C. (2023). Crosstalk between Platelets and SARS-CoV-2: Implications in Thrombo-Inflammatory Complications in COVID-19. Int. J. Mol. Sci..

[17] Al-Kuraishy H.M., Al-Gareeb A.I., Onohuean H., El-Saber Batiha G. (2022). COVID-19 and erythrocrine function: The roller coaster and danger. Int. J. Immunopathol. Pharmacol..

[18] Cosic I., Cosic D., Loncarevic I. (2020). RRM Prediction of Erythrocyte Band3 Protein as Alternative Receptor for SARS-CoV-2 Virus. Appl. Sci..

[19] Behl T., Kaur I., Aleya L., Sehgal A., Singh S., Sharma N., Bhatia S., Al-Harrasi A., Bungau S. (2022). CD147-spike protein interaction in COVID-19: Get the ball rolling with a novel receptor and therapeutic target. Sci. Total Environ..

[20] Kronstein-Wiedemann R., Stadtmuller M., Traikov S., Georgi M., Teichert M., Yosef H., Wallenborn J., Karl A., Schutze K., Wagner M. (2022). SARS-CoV-2 Infects Red Blood Cell Progenitors and Dysregulates Hemoglobin and Iron Metabolism. Stem Cell Rev. Rep..

[21] Toro A., Arevalo A., Pereira-Gómez M., Sabater A., Zizzi E., Pascual G., Lage-Vickers S., Porfido J., Achinelli I., Seniuk R. (2023). Coronavirus pathogenesis in mice explains the SARS-CoV-2 multi-organ spread by red blood cells hitch-hiking. medRxiv.

[22] Huerga Encabo H., Grey W., Garcia-Albornoz M., Wood H., Ulferts R., Aramburu I.V., Kulasekararaj A.G., Mufti G., Papayannopoulos V., Beale R. (2021). Human Erythroid Progenitors Are Directly Infected by SARS-CoV-2: Implications for Emerging Erythropoiesis in Severe COVID-19 Patients. Stem Cell Rep..

[23] Shahbaz S., Xu L., Osman M., Sligl W., Shields J., Joyce M., Tyrrell D.L., Oyegbami O., Elahi S. (2021). Erythroid precursors and progenitors suppress adaptive immunity and get invaded by SARS-CoV-2. Stem Cell Rep..

[24] Bernardes J.P., Mishra N., Tran F., Bahmer T., Best L., Blase J.I., Bordoni D., Franzenburg J., Geisen U., Josephs-Spaulding J. (2020). Longitudinal Multi-omics Analyses Identify Responses of Megakaryocytes, Erythroid Cells, and Plasmablasts as Hallmarks of Severe COVID-19. Immunity.

[25] Saito S., Shahbaz S., Sligl W., Osman M., Tyrrell D.L., Elahi S. (2022). Differential Impact of SARS-CoV-2 Isolates, Namely, the Wuhan Strain, Delta, and Omicron Variants on Erythropoiesis. Microbiol. Spectr..

[26] Shen L., Chen L., Chi H., Luo L., Ruan J., Zhao X., Jiang Y., Tung T.H., Zhu H., Zhou K. (2023). Parameters and Morphological Changes of Erythrocytes and Platelets of COVID-19 Subjects: A Longitudinal Cohort Study. Infect. Drug Resist..

[27] Prudinnik D.S., Sinauridze E.I., Shakhidzhanov S.S., Bovt E.A., Protsenko D.N., Rumyantsev A.G., Ataullakhanov F.I. (2022). Filterability of Erythrocytes in Patients with COVID-19. Biomolecules.

[28] Ananthaseshan S., Bojakowski K., Sacharczuk M., Poznanski P., Skiba D.S., Prahl Wittberg L., McKenzie J., Szkulmowska A., Berg N., Andziak P. (2022). Red blood cell distribution width is associated with increased interactions of blood cells with vascular wall. Sci. Rep..

[29] Bessonov N., Babushkina E., Golovashchenko S., Tosenberger A., Ataullakhanov F., Panteleev M., Tokarev A., Volpert V. (2013). Numerical simulation of blood flows with non-uniform distribution of erythrocytes and platelets. Russ. J. Numer. Anal. Math. Model..

[30] Aarts P.A., van den Broek S.A., Prins G.W., Kuiken G.D., Sixma J.J., Heethaar R.M. (1988). Blood platelets are concentrated near the wall and red blood cells, in the center in flowing blood. Arteriosclerosis.

[31] Walton B.L., Lehmann M., Skorczewski T., Holle L.A., Beckman J.D., Cribb J.A., Mooberry M.J., Wufsus A.R., Cooley B.C., Homeister J.W. (2017). Elevated hematocrit enhances platelet accumulation following vascular injury. Blood.

[32] Weisel J.W., Litvinov R.I. (2019). Red blood cells: The forgotten player in hemostasis and thrombosis. J. Thromb. Haemost..

[33] Westerman M., Porter J.B. (2016). Red blood cell-derived microparticles: An overview. Blood Cells Mol. Dis..

[34] Ferru E., Pantaleo A., Carta F., Mannu F., Khadjavi A., Gallo V., Ronzoni L., Graziadei G., Cappellini M.D., Turrini F. (2014). Thalassemic erythrocytes release microparticles loaded with hemichromes by redox activation of p72Syk kinase. Haematologica.

[35] Nomura S., Shimizu M. (2015). Clinical significance of procoagulant microparticles. J. Intensive Care.

[36] Bouchla A., Kriebardis A.G., Georgatzakou H.T., Fortis S.P., Thomopoulos T.P., Lekkakou L., Markakis K., Gkotzias D., Panagiotou A., Papageorgiou E.G. (2021). Red Blood Cell Abnormalities as the Mirror of SARS-CoV-2 Disease Severity: A Pilot Study. Front. Physiol..

[37] Barr J.D., Chauhan A.K., Schaeffer G.V., Hansen J.K., Motto D.G. (2013). Red blood cells mediate the onset of thrombosis in the ferric chloride murine model. Blood.

[38] Turitto V.T., Weiss H.J. (1980). Red blood cells: Their dual role in thrombus formation. Science.

[39] Zaid Y., Puhm F., Allaeys I., Naya A., Oudghiri M., Khalki L., Limami Y., Zaid N., Sadki K., Ben El Haj R. (2020). Platelets Can Associate with SARS-Cov-2 RNA and Are Hyperactivated in COVID-19. Circ. Res..

[40] Zhang S., Liu Y., Wang X., Yang L., Li H., Wang Y., Liu M., Zhao X., Xie Y., Yang Y. (2020). SARS-CoV-2 binds platelet ACE2 to enhance thrombosis in COVID-19. J. Hematol. Oncol..

[41] Sahai A., Bhandari R., Koupenova M., Freedman J., Godwin M., McIntyre T., Chung M., Iskandar J.P., Kamran H., Aggarwal A. (2020). SARS-CoV-2 Receptors are Expressed on Human Platelets and the Effect of Aspirin on Clinical Outcomes in COVID-19 Patients. Res. Sq..

[42] Alipoor S.D., Mirsaeidi M. (2022). SARS-CoV-2 cell entry beyond the ACE2 receptor. Mol. Biol. Rep..

[43] Bury L., Camilloni B., Castronari R., Piselli E., Malvestiti M., Borghi M., KuchiBotla H., Falcinelli E., Petito E., Amato F. (2021). Search for SARS-CoV-2 RNA in platelets from COVID-19 patients. Platelets.

[44] Campbell R.A., Boilard E., Rondina M.T. (2021). Is there a role for the ACE2 receptor in SARS-CoV-2 interactions with platelets?. J. Thromb. Haemost..

[45] Manne B.K., Denorme F., Middleton E.A., Portier I., Rowley J.W., Stubben C., Petrey A.C., Tolley N.D., Guo L., Cody M. (2020). Platelet gene expression and function in patients with COVID-19. Blood.

[46] Koupenova M., Corkrey H.A., Vitseva O., Tanriverdi K., Somasundaran M., Liu P., Soofi S., Bhandari R., Godwin M., Parsi K.M. (2021). SARS-CoV-2 Initiates Programmed Cell Death in Platelets. Circ. Res..

[47] Garcia C., Au Duong J., Poette M., Ribes A., Payre B., Memier V., Sie P., Minville V., Voisin S., Payrastre B. (2022). Platelet activation and partial desensitization are associated with viral xenophagy in patients with severe COVID-19. Blood Adv..

[48] Shen S., Zhang J., Fang Y., Lu S., Wu J., Zheng X., Deng F. (2021). SARS-CoV-2 interacts with platelets and megakaryocytes via ACE2-independent mechanism. J. Hematol. Oncol..

[49] Zhu A., Real F., Capron C., Rosenberg A.R., Silvin A., Dunsmore G., Zhu J., Cottoignies-Callamarte A., Masse J.M., Moine P. (2022). Infection of lung megakaryocytes and platelets by SARS-CoV-2 anticipate fatal COVID-19. Cell Mol. Life Sci..

[50] Yakusheva A.A., Butov K.R., Bykov G.A., Zavodszky G., Eckly A., Ataullakhanov F.I., Gachet C., Panteleev M.A., Mangin P.H. (2022). Traumatic vessel injuries initiating hemostasis generate high shear conditions. Blood Adv..

[51] Kaiser R., Escaig R., Nicolai L. (2023). Hemostasis without clot formation-how platelets guard the vasculature in inflammation, infection, and malignancy. Blood.

[52] Etulain J., Schattner M. (2014). Glycobiology of platelet-endothelial cell interactions. Glycobiology.

[53] Martyanov A.A., Balabin F.A., Dunster J.L., Panteleev M.A., Gibbins J.M., Sveshnikova A.N. (2020). Control of Platelet CLEC-2-Mediated Activation by Receptor Clustering and Tyrosine Kinase Signaling. Biophys. J..

[54] Fang J., Sun X., Liu S., Yang P., Lin J., Feng J., Cruz M.A., Dong J.F., Fang Y., Wu J. (2021). Shear Stress Accumulation Enhances von Willebrand Factor-Induced Platelet P-Selectin Translocation in a PI3K/Akt Pathway-Dependent Manner. Front. Cell Dev. Biol..

[55] Andrae J., Gallini R., Betsholtz C. (2008). Role of platelet-derived growth factors in physiology and medicine. Genes. Dev..

[56] Shepard J.M., Moon D.G., Sherman P.F., Weston L.K., Del Vecchio P.J., Minnear F.L., Malik A.B., Kaplan J.E. (1989). Platelets decrease albumin permeability of pulmonary artery endothelial cell monolayers. Microvasc. Res..

[57] Cloutier N., Pare A., Farndale R.W., Schumacher H.R., Nigrovic P.A., Lacroix S., Boilard E. (2012). Platelets can enhance vascular permeability. Blood.

[58] Goerge T., Ho-Tin-Noe B., Carbo C., Benarafa C., Remold-O’Donnell E., Zhao B.Q., Cifuni S.M., Wagner D.D. (2008). Inflammation induces hemorrhage in thrombocytopenia. Blood.

[59] Gaertner F., Ahmad Z., Rosenberger G., Fan S., Nicolai L., Busch B., Yavuz G., Luckner M., Ishikawa-Ankerhold H., Hennel R. (2017). Migrating Platelets Are Mechano-scavengers that Collect and Bundle Bacteria. Cell.

[60] Nicolai L., Schiefelbein K., Lipsky S., Leunig A., Hoffknecht M., Pekayvaz K., Raude B., Marx C., Ehrlich A., Pircher J. (2020). Vascular surveillance by haptotactic blood platelets in inflammation and infection. Nat. Commun..

[61] Lan Y., Dong M., Li Y., Diao Y., Chen Z., Wu Z. (2023). Upregulation of girdin delays endothelial cell apoptosis via promoting engulfment of platelets. Mol. Biol. Rep..

[62] Aggarwal A., Jennings C.L., Manning E., Cameron S.J. (2023). Platelets at the Vessel Wall in Non-Thrombotic Disease. Circ. Res..

[63] Yao Y., Sun W., Sun Q., Jing B., Liu S., Liu X., Shen G., Chen R., Wang H. (2019). Platelet-Derived Exosomal MicroRNA-25-3p Inhibits Coronary Vascular Endothelial Cell Inflammation Through Adam10 via the NF-kappaB Signaling Pathway in ApoE(-/-) Mice. Front. Immunol..

[64] Jin P., Pan Q., Lin Y., Dong Y., Zhu J., Liu T., Zhu W., Cheng B. (2023). Platelets Facilitate Wound Healing by Mitochondrial Transfer and Reducing Oxidative Stress in Endothelial Cells. Oxid. Med. Cell Longev..

[65] French S.L., Butov K.R., Allaeys I., Canas J., Morad G., Davenport P., Laroche A., Trubina N.M., Italiano J.E., Moses M.A. (2020). Platelet-derived extracellular vesicles infiltrate and modify the bone marrow during inflammation. Blood Adv..

[66] Martyanov A.A., Boldova A.E., Stepanyan M.G., An O.I., Gur’ev A.S., Kassina D.V., Volkov A.Y., Balatskiy A.V., Butylin A.A., Karamzin S.S. (2022). Longitudinal multiparametric characterization of platelet dysfunction in COVID-19: Effects of disease severity, anticoagulation therapy and inflammatory status. Thromb. Res..

[67] Taus F., Salvagno G., Cane S., Fava C., Mazzaferri F., Carrara E., Petrova V., Barouni R.M., Dima F., Dalbeni A. (2020). Platelets Promote Thromboinflammation in SARS-CoV-2 Pneumonia. Arter. Thromb. Vasc. Biol..

[68] Comer S.P., Cullivan S., Szklanna P.B., Weiss L., Cullen S., Kelliher S., Smolenski A., Murphy C., Altaie H., Curran J. (2021). COVID-19 induces a hyperactive phenotype in circulating platelets. PLoS Biol..

[69] Leopold V., Pereverzeva L., Schuurman A.R., Reijnders T.D.Y., Saris A., de Brabander J., van Linge C.C.A., Douma R.A., Chouchane O., Nieuwland R. (2021). Platelets are Hyperactivated but Show Reduced Glycoprotein VI Reactivity in COVID-19 Patients. Thromb. Haemost..

[70] Hottz E.D., Azevedo-Quintanilha I.G., Palhinha L., Teixeira L., Barreto E.A., Pao C.R.R., Righy C., Franco S., Souza T.M.L., Kurtz P. (2020). Platelet activation and platelet-monocyte aggregate formation trigger tissue factor expression in patients with severe COVID-19. Blood.

[71] Barrett T.J., Bilaloglu S., Cornwell M., Burgess H.M., Virginio V.W., Drenkova K., Ibrahim H., Yuriditsky E., Aphinyanaphongs Y., Lifshitz M. (2021). Platelets contribute to disease severity in COVID-19. J. Thromb. Haemost..

[72] Dorken-Gallastegi A., Lee Y., Li G., Li H., Naar L., Li X., Ye T., Van Cott E., Rosovsky R., Gregory D. (2023). Circulating cellular clusters are associated with thrombotic complications and clinical outcomes in COVID-19. iScience.

[73] Malengier-Devlies B., Filtjens J., Ahmadzadeh K., Boeckx B., Vandenhaute J., De Visscher A., Bernaerts E., Mitera T., Jacobs C., Vanderbeke L. (2022). Severe COVID-19 patients display hyper-activated NK cells and NK cell-platelet aggregates. Front. Immunol..

[74] Srihirun S., Sriwantana T., Srichatrapimuk S., Vivithanaporn P., Kirdlarp S., Sungkanuparph S., Phusanti S., Nanthatanti N., Suwannalert P., Sibmooh N. (2023). Increased platelet activation and lower platelet-monocyte aggregates in COVID-19 patients with severe pneumonia. PLoS ONE.

[75] Delshad M., Safaroghli-Azar A., Pourbagheri-Sigaroodi A., Poopak B., Shokouhi S., Bashash D. (2021). Platelets in the perspective of COVID-19; pathophysiology of thrombocytopenia and its implication as prognostic and therapeutic opportunity. Int. Immunopharmacol..

[76] Ignatova A.A., Demina I.A., Ptushkin V.V., Khaspekova S.G., Shustova O.N., Pankrashkina M.M., Ryabykh A.A., Obydennyi S.I., Strelkova O.S., Polokhov D.M. (2019). Evolution of platelet function in adult patients with chronic immune thrombocytopenia on romiplostim treatment. Br. J. Haematol..

[77] Frelinger A.L., Grace R.F., Gerrits A.J., Berny-Lang M.A., Brown T., Carmichael S.L., Neufeld E.J., Michelson A.D. (2015). Platelet function tests, independent of platelet count, are associated with bleeding severity in ITP. Blood.

[78] Frelinger A.L., Grace R.F., Gerrits A.J., Carmichael S.L., Forde E.E., Michelson A.D. (2018). Platelet Function in ITP, Independent of Platelet Count, Is Consistent Over Time and Is Associated with Both Current and Subsequent Bleeding Severity. Thromb. Haemost..

[79] Panzer S., Rieger M., Vormittag R., Eichelberger B., Dunkler D., Pabinger I. (2007). Platelet function to estimate the bleeding risk in autoimmune thrombocytopenia. Eur. J. Clin. Investig..

[80] Nishiura N., Kashiwagi H., Akuta K., Hayashi S., Kato H., Kanakura Y., Tomiyama Y. (2020). Reevaluation of platelet function in chronic immune thrombocytopenia: Impacts of platelet size, platelet-associated anti-alphaIIbbeta3 antibodies and thrombopoietin receptor agonists. Br. J. Haematol..

[81] van Bladel E.R., Laarhoven A.G., van der Heijden L.B., Heitink-Polle K.M., Porcelijn L., van der Schoot C.E., de Haas M., Roest M., Vidarsson G., de Groot P.G. (2014). Functional platelet defects in children with severe chronic ITP as tested with 2 novel assays applicable for low platelet counts. Blood.

[82] Van Den Helm S., Yaw H.P., Letunica N., Barton R., Weaver A., Newall F., Horton S.B., Chiletti R., Johansen A., Best D. (2022). Platelet Phenotype and Function Changes With Increasing Duration of Extracorporeal Membrane Oxygenation. Crit. Care Med..

[83] Cheung P.Y., Sawicki G., Salas E., Etches P.C., Schulz R., Radomski M.W. (2000). The mechanisms of platelet dysfunction during extracorporeal membrane oxygenation in critically ill neonates. Crit. Care Med..

[84] Stallion A., Cofer B.R., Rafferty J.A., Ziegler M.M., Ryckman F.C. (1994). The significant relationship between platelet count and haemorrhagic complications on ECMO. Perfusion.

[85] Negrut N., Codrean A., Hodisan I., Bungau S., Tit D.M., Marin R., Behl T., Banica F., Diaconu C.C., Nistor-Cseppento D.C. (2021). Efficiency of antiviral treatment in COVID-19. Exp. Ther. Med..

[86] Pontolillo M., Ucciferri C., Borrelli P., Di Nicola M., Vecchiet J., Falasca K. (2022). Molnupiravir as an Early Treatment for COVID-19: A Real Life Study. Pathogens.

[87] Fischer A.L., Messer S., Riera R., Martimbianco A.L.C., Stegemann M., Estcourt L.J., Weibel S., Monsef I., Andreas M., Pacheco R.L. (2023). Antiplatelet agents for the treatment of adults with COVID-19. Cochrane Database Syst. Rev..

[88] Artemenko E.O., Yakimenko A.O., Pichugin A.V., Ataullakhanov F.I., Panteleev M.A. (2016). Calpain-controlled detachment of major glycoproteins from the cytoskeleton regulates adhesive properties of activated phosphatidylserine-positive platelets. Biochem. J..

[89] Sveshnikova A.N., Ataullakhanov F.I., Panteleev M.A. (2015). Compartmentalized calcium signaling triggers subpopulation formation upon platelet activation through PAR1. Mol. Biosyst..

[90] Obydennyy S.I., Sveshnikova A.N., Ataullakhanov F.I., Panteleev M.A. (2016). Dynamics of calcium spiking, mitochondrial collapse and phosphatidylserine exposure in platelet subpopulations during activation. J. Thromb. Haemost..

[91] Obydennyi S.I., Artemenko E.O., Sveshnikova A.N., Ignatova A.A., Varlamova T.V., Gambaryan S., Lomakina G.Y., Ugarova N.N., Kireev I.I., Ataullakhanov F.I. (2020). Mechanisms of increased mitochondria-dependent necrosis in Wiskott-Aldrich syndrome platelets. Haematologica.

[92] Abaeva A.A., Canault M., Kotova Y.N., Obydennyy S.I., Yakimenko A.O., Podoplelova N.A., Kolyadko V.N., Chambost H., Mazurov A.V., Ataullakhanov F.I. (2013). Procoagulant platelets form an alpha-granule protein-covered “cap” on their surface that promotes their attachment to aggregates. J. Biol. Chem..

[93] Yakimenko A.O., Verholomova F.Y., Kotova Y.N., Ataullakhanov F.I., Panteleev M.A. (2012). Identification of different proaggregatory abilities of activated platelet subpopulations. Biophys. J..

[94] Morozova D.S., Martyanov A.A., Obydennyi S.I., Korobkin J.D., Sokolov A.V., Shamova E.V., Gorudko I.V., Khoreva A.L., Shcherbina A., Panteleev M.A. (2022). Ex vivo observation of granulocyte activity during thrombus formation. BMC Biol..

[95] Koltsova E.M., Balashova E.N., Ignatova A.A., Poletaev A.V., Polokhov D.M., Kuprash A.D., Ionov O.V., Kirtbaya A.R., Lenyushkina A.A., Timofeeva L.A. (2019). Impaired platelet activity and hypercoagulation in healthy term and moderately preterm newborns during the early neonatal period. Pediatr. Res..

[96] Althaus K., Marini I., Zlamal J., Pelzl L., Singh A., Haberle H., Mehrlander M., Hammer S., Schulze H., Bitzer M. (2021). Antibody-induced procoagulant platelets in severe COVID-19 infection. Blood.

[97] Claude L., Martino F., Hermand P., Chahim B., Roger P.M., de Bourayne M., Garnier Y., Tressieres B., Colin Y., Le Van Kim C. (2022). Platelet caspase-1 and Bruton tyrosine kinase activation in patients with COVID-19 is associated with disease severity and reversed in vitro by ibrutinib. Res. Pr. Thromb. Haemost..

[98] Uzun G., Singh A., Abou-Khalel W., Pelzl L., Weich K., Nowak-Harnau S., Althaus K., Bugert P., Kluter H., Bakchoul T. (2022). Platelets and Sera from Donors of Convalescent Plasma after Mild COVID-19 Show No Procoagulant Phenotype. Hamostaseologie.

[99] Ignatova A.A., Ponomarenko E.A., Polokhov D.M., Suntsova E.V., Zharkov P.A., Fedorova D.V., Balashova E.N., Rudneva A.E., Ptushkin V.V., Nikitin E.A. (2019). Flow cytometry for pediatric platelets. Platelets.

[100] Colicchia M., Schrottmaier W.C., Perrella G., Reyat J.S., Begum J., Slater A., Price J., Clark J.C., Zhi Z., Simpson M.J. (2022). S100A8/A9 drives the formation of procoagulant platelets through GPIbalpha. Blood.

[101] Cappelletto A., Allan H.E., Crescente M., Schneider E., Bussani R., Ali H., Secco I., Vodret S., Simeone R., Mascaretti L. (2022). SARS-CoV-2 Spike protein activates TMEM16F-mediated platelet procoagulant activity. Front. Cardiovasc. Med..

[102] Gabanella F., Barbato C., Corbi N., Fiore M., Petrella C., de Vincentiis M., Greco A., Ferraguti G., Corsi A., Ralli M. (2022). Exploring Mitochondrial Localization of SARS-CoV-2 RNA by Padlock Assay: A Pilot Study in Human Placenta. Int. J. Mol. Sci..

[103] Wu K.E., Fazal F.M., Parker K.R., Zou J., Chang H.Y. (2020). RNA-GPS Predicts SARS-CoV-2 RNA Residency to Host Mitochondria and Nucleolus. Cell Syst..

[104] Valdes-Aguayo J.J., Garza-Veloz I., Badillo-Almaraz J.I., Bernal-Silva S., Martinez-Vazquez M.C., Juarez-Alcala V., Vargas-Rodriguez J.R., Gaeta-Velasco M.L., Gonzalez-Fuentes C., Avila-Carrasco L. (2021). Mitochondria and Mitochondrial DNA: Key Elements in the Pathogenesis and Exacerbation of the Inflammatory State Caused by COVID-19. Medicina.

[105] Srinivasan K., Pandey A.K., Livingston A., Venkatesh S. (2021). Roles of host mitochondria in the development of COVID-19 pathology: Could mitochondria be a potential therapeutic target?. Mol. Biomed..

[106] Bhowal C., Ghosh S., Ghatak D., De R. (2023). Pathophysiological involvement of host mitochondria in SARS-CoV-2 infection that causes COVID-19: A comprehensive evidential insight. Mol. Cell Biochem..

[107] Sumbalova Z., Kucharska J., Palacka P., Rausova Z., Langsjoen P.H., Langsjoen A.M., Gvozdjakova A. (2022). Platelet mitochondrial function and endogenous coenzyme Q10 levels are reduced in patients after COVID-19. Bratisl. Lek. Listy.

[108] De la Cruz-Enriquez J., Rojas-Morales E., Ruiz-Garcia M.G., Tobon-Velasco J.C., Jimenez-Ortega J.C. (2021). SARS-CoV-2 induces mitochondrial dysfunction and cell death by oxidative stress/inflammation in leukocytes of COVID-19 patients. Free Radic. Res..

[109] Pliss A., Kuzmin A.N., Prasad P.N., Mahajan S.D. (2022). Mitochondrial Dysfunction: A Prelude to Neuropathogenesis of SARS-CoV-2. ACS Chem. Neurosci..

[110] Huynh T.V., Rethi L., Lee T.W., Higa S., Kao Y.H., Chen Y.J. (2023). Spike Protein Impairs Mitochondrial Function in Human Cardiomyocytes: Mechanisms Underlying Cardiac Injury in COVID-19. Cells.

[111] Mykytyn A.Z., Fouchier R.A., Haagmans B.L. (2023). Antigenic evolution of SARS coronavirus 2. Curr. Opin. Virol..

[112] Middleton C., Kubatko L. (2023). Assessment of positive selection across SARS-CoV-2 variants via maximum likelihood. PLoS ONE.

[113] Volz E. (2023). Fitness, growth and transmissibility of SARS-CoV-2 genetic variants. Nat. Rev. Genet..

[114] Wolf J.M., Wolf L.M., Bello G.L., Maccari J.G., Nasi L.A. (2023). Molecular evolution of SARS-CoV-2 from December 2019 to August 2022. J. Med. Virol..

[115] Akkiz H. (2022). The Biological Functions and Clinical Significance of SARS-CoV-2 Variants of Corcern. Front Med..

[116] Bhatia S., Wardle J., Nash R.K., Nouvellet P., Cori A. (2023). Extending EpiEstim to estimate the transmission advantage of pathogen variants in real-time: SARS-CoV-2 as a case-study. Epidemics.

[117] Behl T., Kaur I., Sehgal A., Singh S., Sharma N., Anwer M.K., Makeen H.A., Albratty M., Alhazmi H.A., Bhatia S. (2022). There is nothing exempt from the peril of mutation-The Omicron spike. Biomed. Pharmacother..

[118] Krygier A., Szmajda-Krygier D., Swiechowski R., Pietrzak J., Wosiak A., Wodzinski D., Balcerczak E. (2022). Molecular Pathogenesis of Fibrosis, Thrombosis and Surfactant Dysfunction in the Lungs of Severe COVID-19 Patients. Biomolecules.

[119] Manzur-Pineda K., O’Neil C.F., Bornak A., Lalama M.J., Shao T., Kang N., Kennel-Pierre S., Tabbara M., Velazquez O.C., Rey J. (2022). COVID-19-related thrombotic complications experience before and during delta wave. J. Vasc. Surg..

[120] Hungaro Cunha C., Yuri Sato D., Pereira de Godoy J.M., da Silva Russeff G.J., Franccini Del Frari Silva D., Pereira de Godoy H.J., Menezes da Silva M.O., Amorim Santos H., Guerreiro Godoy M.F. (2022). Mortality and Deep Vein Thrombosis in the Gamma Variant of Covid 19 and Lung Injury. Vasc. Health Risk Manag..

[121] Xie B., Semaan D.B., Binko M.A., Agrawal N., Kulkarni R.N., Andraska E.A., Sachdev U., Chaer R.A., Eslami M.H., Makaroun M.S. (2023). COVID-associated acute limb ischemia during the Delta surge and the effect of vaccines. J. Vasc. Surg..

[122] Bhandari S., Joshi S., Rankawat G., Tiwaskar M., Lohmror A., Singh A. (2023). Post-COVID-19 Arterial Thrombotic Events among Three Major Permutations of COVID-19. J. Assoc. Physicians India.

[123] Ma X., Liang J., Zhu G., Bhoria P., Shoara A.A., MacKeigan D.T., Khoury C.J., Slavkovic S., Lin L., Karakas D. (2023). SARS-CoV-2 RBD and Its Variants Can Induce Platelet Activation and Clearance: Implications for Antibody Therapy and Vaccinations against COVID-19. Research.

[124] Ito K., Goto K., Shirakawa R., Horiuchi H., Ogasawara K. (2023). Platelet alphaIIbbeta3 integrin binds to SARS-CoV-2 spike protein of alpha strain but not wild type and omicron strains. Biochem. Biophys. Res. Commun..

[125] Kusudo E., Murata Y., Kawamoto S., Egi M. (2023). Variant-derived SARS-CoV-2 spike protein does not directly cause platelet activation or hypercoagulability. Clin. Exp. Med..

[126] Vettori M., Carpene G., Salvagno G.L., Gelati M., Dima F., Celegon G., Favaloro E.J., Lippi G. (2023). Effects of Recombinant SARS-CoV-2 Spike Protein Variants on Platelet Morphology and Activation. Seminars in Thrombosis and Hemostasis.

[127] Sevilya Z., Kuzmina A., Cipok M., Hershkovitz V., Keidar-Friedman D., Taube R., Lev E.I. (2023). Differential platelet activation through an interaction with spike proteins of different SARS-CoV-2 variants. J. Thromb. Thrombolysis.

[128] Taylor F.B. (1994). Studies on the inflammatory-coagulant axis in the baboon response to E. coli: Regulatory roles of proteins C, S, C4bBP and of inhibitors of tissue factor. Prog. Clin. Biol. Res..

[129] Bernard I., Limonta D., Mahal L.K., Hobman T.C. (2020). Endothelium Infection and Dysregulation by SARS-CoV-2: Evidence and Caveats in COVID-19. Viruses.

[130] Castanheira F.V.S., Nguyen R., Willson M., Davoli-Ferreira M., David B.A., Kelly M.M., Lee W.Y., Kratofil R.M., Zhang W.X., Bui-Marinos M. (2023). Intravital imaging of three different microvascular beds in SARS-CoV-2-infected mice. Blood Adv..

[131] Colicchia M., Perrella G., Gant P., Rayes J. (2023). Novel mechanisms of thrombo-inflammation during infection: Spotlight on neutrophil extracellular trap-mediated platelet activation. Res. Pr. Thromb. Haemost..

[132] Lin H., Liu J., Li N., Zhang B., Nguyen V.D., Yao P., Feng J., Liu Q., Chen Y., Li G. (2023). NETosis promotes chronic inflammation and fibrosis in systemic lupus erythematosus and COVID-19. Clin. Immunol..

[133] Krinsky N., Sizikov S., Nissim S., Dror A., Sas A., Prinz H., Pri-Or E., Perek S., Raz-Pasteur A., Lejbkowicz I. (2023). NETosis induction reflects COVID-19 severity and long COVID: Insights from a 2-center patient cohort study in Israel. J. Thromb. Haemost..

[134] Bhargavan B., Kanmogne G.D. (2023). SARS-CoV-2 Spike Proteins and Cell-Cell Communication Induce P-Selectin and Markers of Endothelial Injury, NETosis, and Inflammation in Human Lung Microvascular Endothelial Cells and Neutrophils: Implications for the Pathogenesis of COVID-19 Coagulopathy. Int. J. Mol. Sci..

[135] Park C., Hwang I.Y., Yan S.L., Vimonpatranon S., Wei D., Van Ryk D., Cicala C., Arthos J., Kehrl J.H. (2023). Murine Alveolar Macrophages Rapidly Accumulate Intranasally Administered SARS-CoV-2 Spike Protein leading to Neutrophil Recruitment and Damage. bioRxiv.

[136] Zakharova N.V., Artemenko E.O., Podoplelova N.A., Sveshnikova A.N., Demina I.A., Ataullakhanov F.I., Panteleev M.A. (2015). Platelet surface-associated activation and secretion-mediated inhibition of coagulation factor XII. PLoS ONE.

[137] Sciaudone A., Corkrey H., Humphries F., Koupenova M. (2023). Platelets and SARS-CoV-2 During COVID-19: Immunity, Thrombosis, and Beyond. Circ. Res..

